# Localization and Distribution of Testicular Angiotensin I Converting Enzyme (ACE) in Neck and Mid-Piece of Spermatozoa from Infertile Men in Relation to Sperm Motility

**DOI:** 10.3390/cells10123572

**Published:** 2021-12-17

**Authors:** Mina Pencheva, Donka Keskinova, Pavel Rashev, Yvetta Koeva, Nina Atanassova

**Affiliations:** 1 Department of Medical Physics and Biophysics, Faculty of Pharmacy, Medical University Plovdiv, 4002 Plovdiv, Bulgaria; 2 Department of Applied and Institutional Sociology, Faculty of Philosophy and History, University of Plovdiv Paisii Hilendarski, 4000 Plovdiv, Bulgaria; d.keskinova@uni-plovdiv.bg; 3 Institute of Biology and Immunology of Reproduction “Akad. K. Bratanov”, Bulgarian Academy of Sciences, 1113 Sofia, Bulgaria; pavel_rashev@abv.bg; 4 Department of Anatomy, Histology and Embryology, Medical University of Plovdiv, 4002 Plovdiv, Bulgaria; Iveta.Koeva@mu-plovdiv.bg; 5 Institute of Experimental Morphology, Pathology and Anthropology with Museum, Bulgarian Academy of Sciences, 1113 Sofia, Bulgaria; ninaatanassova@yahoo.com

**Keywords:** tACE, sperm, capacitation, acrosome reaction, male infertility

## Abstract

Testicular angiotensin converting enzyme (ACE) is known to play an essential role in the male reproduction and fertility. Data about tACE in cases of male infertility are quite scarce, and in this respect we aimed to study localization and distribution of tACE protein in the neck and mid-piece of spermatozoa from pathological samples in relation to sperm motility. The enzyme expression during capacitation and acrosome reaction was quantitatively assessed. In human ejaculated spermatozoa tACE is localized on sperm plasma membrane of the head, the neck and mid-piece of the tail. The immunoreactivity becomes stronger in capacitated spermatozoa followed by a decrease in acrosome reacted sperm. In different cases of semen pathology (oligozoospermia, asthenozoospermia and teratozoospermia) fluorescent signals in the neck and mid-piece are in punctate manner whereas in normozoospermia they were uniformly distributed. The expression area of tACE the neck and mid-piece was decreased in ejaculated and capacitated sperm from pathological semen samples compared to normospermia. Significant positive correlation was established between tACE area and progressive sperm motility, whereas with immotile sperm the correlation was negative. Our data suggest that proper distribution of tACE in the neck and mid-piece is required for normal sperm motility that could be used as a novel biomarker for male infertility.

## 1. Introduction

Infertility affects an estimated 15% of couples globally, amounting to 48.5 million couples. Problems in male’ reproductive systems are found to be solely responsible for 20–30% of infertility cases and contribute to 50% of cases overall [1]. Diagnosis in men consists of semen analysis, but in a large percentage of cases it is not sufficient to determine the complex etiological causes leading to idiopathic infertility [2]. In these patients, the use of assisted reproductive techniques such as ICSI (intracytoplasmic sperm injection) is suggested as a solution, but the choice of sperm with good quality remains a problem. A huge number of enzymes are found on the sperm membrane, involved in complex signaling pathways, ensuring the ability of sperm to go the long way from testicular differentiation, maturation and capacitation, hyper-activation to fusion with the egg and embryo development.

One of the enzymes that has been shown to be important in male reproduction and fertility is angiotensin I—converting enzyme (ACE)—an important component of renin-angiotensin system (RAS) ACE is a membrane bound Zn2+ metalloproteinase dipeptidyl carboxypeptidase that removes two residues from C terminus of certain peptides [3].

ACE exists in two isoforms—somatic (sACE) and testis-specific (tACE)—and both are encoded by the same gene. The testicular isoform of ACE is expressed only in the testis during development of germ cells being localized in haploid elongating spermatids and spermatozoa [4]. Somatic ACE is secreted by epithelial cells of male reproductive tract being a component of seminal plasma [5].

For better understanding of the role of testicular and somatic isoforms of ACE in male reproduction, an insertional disruption of somatic but not testicular ACE gene was generated [6]. Males homozygous for sACE mutation have normal fertility, proving conclusively that somatic ACE is not essential for their fertility. ACE null mice lacking both somatic and testicular ACE are infertile due to altered migration of sperm in the oviduct and their inability to bind zona pellucida suggesting that only tACE has critical importance for male fertility. Experiments with transgenic expression of tACE in ACE null mice restored fertility, whereas transgenesis of sACE in ACE mutants did not and mice are infertile. Therefore, sACE cannot substitute tACE in male reproduction [7].

Testicular ACE acts as dipeptidase and as a GPI-anchored protein releasing factor and both activities are of great importance for fertilizing ability of spermatozoa [8,9]. As a dipeptidase, tACE acts in the sperm on epididymal transit, whereas another enzymatic activity, performed in female reproductive tract, is responsible for shedding of various GPI-anchored proteins from the cell surface of germ cells, necessary for sperm–zona pellucida binding. Hence, tACE may serve as marker for fertilizing ability of spermatozoa. Recent studies by Gianzo et al. (2018) [10] suggested that tACE could be used in selecting better semen samples for obtaining high quality embryo during IVF procedure.

Motility is one of the main characteristic features, considered as a “quality factor” of spermatozoa. Movement of sperms is performed by a mitochondrial sheath, located in the mid-piece of sperm tail. Sperm motility is dependent on complex orchestrated biological systems including RAS, of which the components angiotensin I, angiotensin II and ACE are known to be localized in male reproductive organs. Angiotensin II has been reported to directly stimulate sperm motility [11].

Most of the data about the role of ACE in male reproduction originate from animal experimental models. Studies by Pauls et al. (2003 [12]) reported localization of tACE in post-meiotic spermatids and at the neck and midpiece region of ejaculated human spermatozoa. Earlier studies by Kohn et al. (1998 [13]) found ACE on sperm head, mid-piece and flagellum. However, data about localization and distribution of tACE in cases of male infertility are rather scarce.

So far, few quantitative studies were done involving measurements of enzyme activity or amount of membrane bound tACE in infertile patients that negatively correlated with sperm motility [13,14,15]. The level of tACE expression on the surface of ejaculated spermatozoa is of great importance for fertilization and it has clinical significance for diagnostic of male infertility [16]. In human sperm, release of tACE during capacitation is independent of acrosome reaction and measurement of tACE release was proposed as a clinical assay for human sperm capacitation [17] There is lack of data about expression of tACE during maturational changes in spermatozoa in cases of male infertility.

In this respect the aim of the present paper was to study localization and distribution of tACE protein on sperm tail (neck and mid-piece) in different categories of infertile men in relation to sperm motility. Moreover, dynamic of tACE protein immunoreactivity in the course of capacitation and acrosome reaction was evaluated in different cases of male infertility. In this regard the current study will provide new knowledge about potential use of tACE as a biomarker for motility and fertilizing ability of spermatozoa.

## 2. Materials and Methods

### 2.1. Sperm Analysis

A total of 111 patients who visited the specialized in vitro hospital in 2017–2018 participated in the study. Each patient had to fill a number of documents according to the requirements.

All the patients participating in the study were aged between 20 and 51 years. Semen samples were collected by masturbation, after 3–5 days of sexual abstinence, in a sterile container, and stored at room temperature (18–20 °C). After complete liquefaction, the ejaculates were analyzed and classified as normospermic according to the standards of the World Health Organization [18] when: sperm concentration in 1 mL (≥15 million/mL) and in the entire ejaculate (≥39 million/ejaculate), sperm motility (progressive + non-progressive ≥ 40%), progressively motile sperm (PR ≥ 32%) and sperm morphology (≥4%).

The ejaculate was subjected to Computer-Assisted Sperm Analysis (CASA, Microptic, Barcelona, Spain) for measurement of total sperm count, sperm concentration (cells × 10^6^/^mL^) and sperm motility (progressive and non-progressive). The number of progressive, non-progressive and immotile sperm were expressed as a percentage of the total number of sperm. The semen was loaded into a Leja 20 chamber (Leja Products B.V., Nieuw-Vennep, The Netherlands) and examined using a microscope (Nikon, Tokyo, Japan) with a warm stage at 37 °C

For morphological assessment semen was washed with FertiCult Flushing medium, Fertipro (Beernem, Belgium) and centrifuged at 300 *g* for 10 min. The supernatant was removed and 0.5 mL of FertiCult medium was added to the remaining pellet. Ten microliters of washed semen was then spread onto a glass slide, fixed and air-dried. Smears were stained with Spermac stain, FertiPro (Beernem, Belgium). At least 200 cells were counted at 100 × objective fitted to Zeiss AxioScope A1. For morphological assessment Kruger strict criteria were applied [19]

Normal and pathologic semen samples were evaluated according to WHO standards (WHO, 2010 [18]): normozoospermic (*n* = 31 men), teratozoospermic (*n* = 24 patients), asthenozoospermic (*n* = 31 patients) or oligozoospermic (*n* = 25 patients).

### 2.2. Processing of Ejaculate

**Figure 1:**
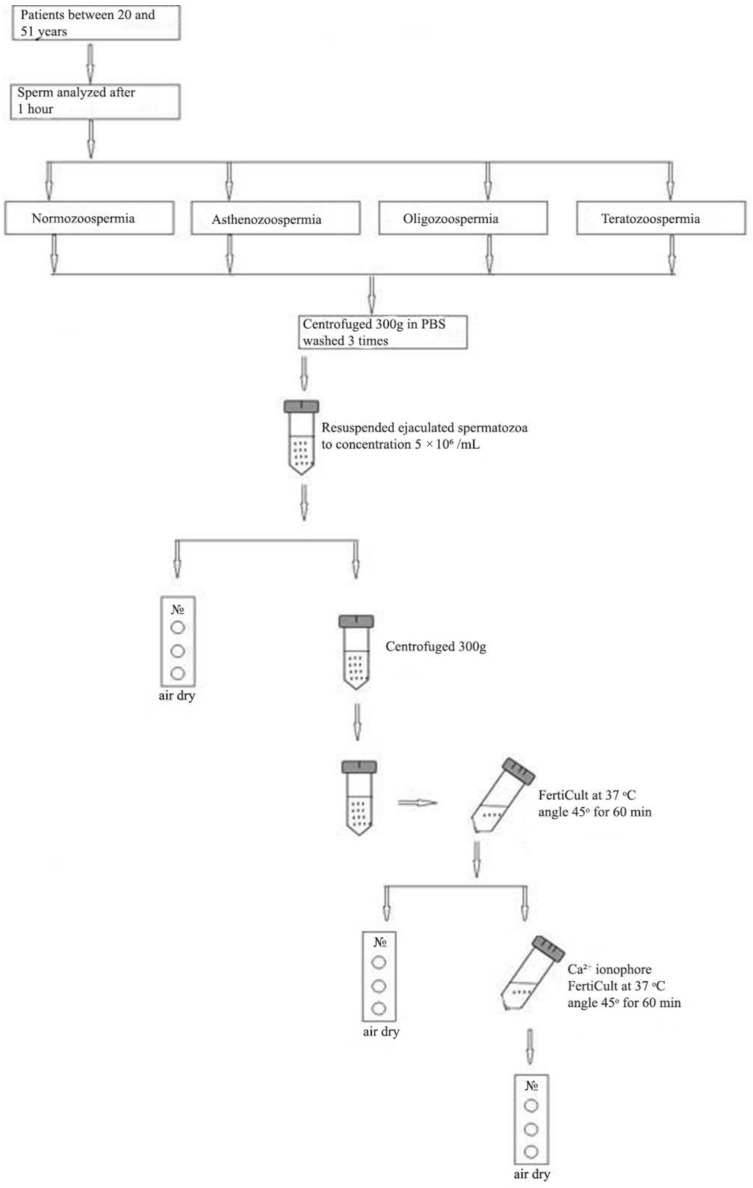
Schematic representation of sperm processing.

The ejaculated spermatozoa were washed three times with PBS, re-suspended, centrifuged at 300× *g* and the supernatants were discarded (schematic presentation is shown on Figure 1). Pellet was re-suspended in PBS to a concentration of 5 × 10^6^ cells/mL. One part from untreated ejaculated sperm was used immediately after the end of the procedure to drop 20 μL on the slides and allowed them to air dry. Second part form untreated ejaculated sperm was centrifuged at 300 g and capacitation medium (FertiCult, Ferti Pro, Belgium) was added to the pellet. Then placed for 1 h in an incubator at 37 °C, 5% CO2 in order to reach the surface by means of the Swim-up method and 20 μL from the surface fraction were taken, dropped on the slides and air-dried. For sperm capacitation from normal and pathological semen samples the protocol by Gianzo et al., 2016 was applied [20]. Calcium ionophore A23187 (sc-3591, Santa Cruz Biotechnology, Inc., Texas, USA) was added in final concentration 20 μM to stimulate acrosome reaction and placed back in an incubator for 1 h. Then the samples were removed from the incubator and material was taken from the surface layer, 20 μL were dropped on glass slides and air dried. For fixation, ice methanol was applied for 20 min and then air-dried. The fixed material was stored in a refrigerator at +4 °C for up to 3 months. All slides used were coated with an adhesive (Poly-L-lysine, Sigma Chemical Co., St. Louis, MO, USA).

### 2.3. Immunofluorescence

Sperm smears were prepared for every in vitro incubation time stated above. Sperm were washed three times in PBS, smeared onto glass slides and air-dried. Sperm smears were fixed with 4% paraformaldehyde in PBS, treated of 0.5% Triton X-100 for 10 min, followed by washing in 3 xPBS. Sperm were blocked with 10% BSA in PBS for 1 h and incubated with primary antibody ACE (sc-12187, Santa Cruz Biotechnology, Inc) diluted 1:500 in 1% BSA in PBS overnight at 4 °C, followed by incubation with goat anti-rabbit IgG Alexa Fluor-568, (ab-175470, Abcam). After washing, the slides were mounted using a Vectashield mounting medium with DAPI (Vector Lab., Burlingame, CA, USA). The samples were examined with laser scanning confocal microscope Leica TCS SPE (Leica Microsystems GmbH, 35578 Wetzlar, Germany).

The primary antibody used in the current study detects both isoforms sACE and tACE. According to the several authors three washes of spermatozoa are sufficient to remove sACE from the surface of spermatozoa without any loss of tACE [21,22,23]. In this way cross-reactivity between two isoformof ACE is avoided. The specificity of the antibody used in the current study (sc-12187, Santa Cruz Biotechnology, Inc) was previously validated by Western blot by Li et al., 2014 [23]. In the current study, spermatozoa were washed three times (during processing of ejaculate) before they were used for immunofluorescence of ejaculated spermatozoa or before they were treated for capacitation. Negative controls were run in parallel by omitting of the primary antibody under the same conditions.

To determine the functional status of spermatozoa (ejaculated, capacitated and acrosome-reacted), staining with Pisum sativum agglutinin (PSA)-FITC was used.

### 2.4. Morphometric Analysis of Sperm

The assay was performed on slides with sperm smears using an indirect immunofluorescence method, describe above, in which the nucleus was stained blue (DAPI) and red fluorescent signals of anti-ACE antibody binding on spermatozoa. For sperm microphotography we used an x ×100 objective of a Leica TCS SPE confocal microscope (Leica Microsystems GmbH, 35578 Wetzlar Germany), equipped with four solid-state lasers with wavelengths from 488 to 635 nm and Leica ACS (Advanced Correction System) technology for perfect colocalization and maximum transmission. The digitized micrographs with 262,144 pixels (picture elements) and 256 levels of gray were used to analyze the image of the sperm using Olympus DP—Soft 4.1 software, Japan. The cells were randomly selected for morphometric analysis, and the computer software used an area of μm^2^ to measure the size of the tACE visualization in the neck and middle piece sperm of the four groups, and the data were recorded in Excel^®^ (Microsoft^®^ Corporation, Redmond, Washington, USA). Measurements of tACE area were done on at least 100 cells from each patient group (normospermia, oligozoospermia, asthenozoospermia and teratozoospermia) in each condition (ejaculated, capacitated and acrosome reacted sperm).

### 2.5. Statistical Analysis

Statistical analysis was carried out using IBM SPSS Statistics (v25) and significance was fixed at *p* < 0.05. All indicators were presented as mean and standard deviation (mean ± SD). The group means by indicator were compared using a *t*-test or Mann-Whitney test for two independent samples, depending on the presence or absence of a normal distribution of indicators established by Kolmogorov–Smirnov test with Lilliefors Significance Correction. The means for tACE area in untreated, capacitated and acrosome reacted sperm in each group were compared with the Paired Samples Test or Wilcoxon Signed Ranks Test for two dependent samples, depending on the form of distribution. Correlation analysis was used to study relationships between the individual indicators of sperm analysis and the area of expression of ACE in the neck and mid piece. Due to the absence of a normal distribution in most indicators and the heteroscedasticity of the points in the scatter plot, the nonparametric Spearman rank coefficient was used.

## 3. Results

The sperm concentration in pathological samples was lower compared to normospermia and in oligozoospermia the parameter was quite lower than asthenozoospermia and teratozoospermia (Table 1). Percentage of progressive, non-progressive and immotile sperm were assessed in normospermic and in pathological semen samples. Progressive sperm motility of pathological samples was significantly lower than normospermic men and the lowest value is found in asthenozoospermic men. Percentage of immotile sperm was higher in pathological semen samples and the highest value is found in asthenozoospermic men. Higher percentage of sperm with defects in the neck and mid-piece was established in pathological samples and the highest value is found in teratozoospermia.

### 3.1. Expression of tACE Protein in Ejaculated, Capacitated and Acrosome Reacted Spermatozoa in Different Cases of Male Infertility

**Figure 2:**
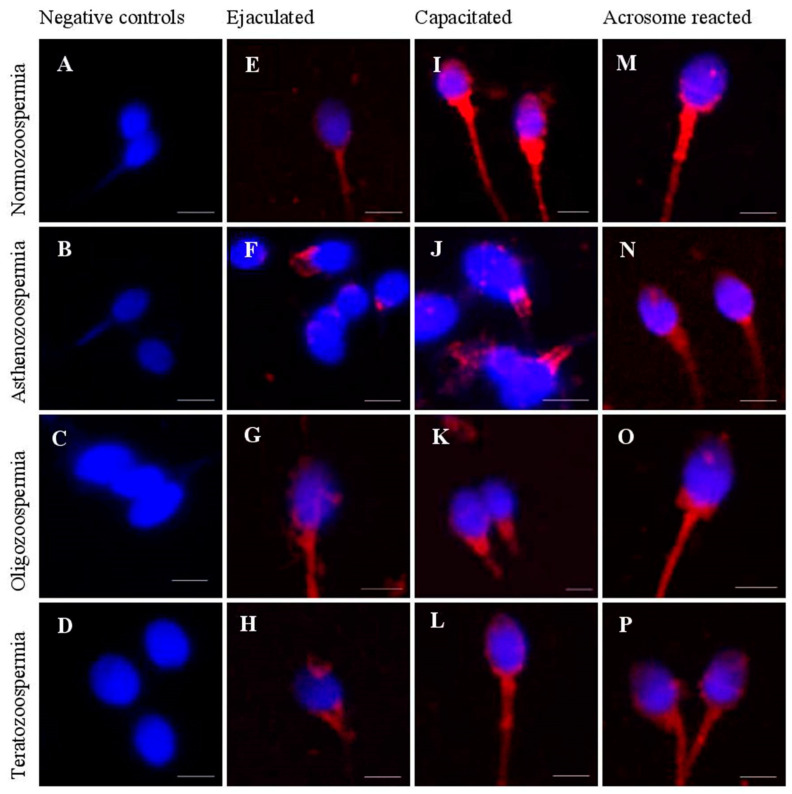
Indirect immunofluorescent of tACE protein expression in ejaculated (**E**–**H**) capacitated (**I**–**L**) and acrosome reacted (**M**–**P**) sperm from different cases of semen pathology (asthenozoospermia, oligozoospermia and teratozoospermia). Note punctate pattern of distribution of tACE in the neck and mid-piece compared to uniform distribution in normospermia. The panel (**A**–**D**) represents negative controls by omitting of primary antibody in samples from ejaculated spermatozoa from normospermia and from different cases of semen pathology (asthenozoospermia, oligozoospermia and teratozoospermia). Scale bar = 5 μm.

Our observation based on immunofluorescence revealed that in ejaculated spermatozoa from normospermic men the tACE protein expression was spread on the plasma membrane of sperm head. In the neck and mid-piece of the tail, tACE was distributed uniformly whereas in pathological semen samples (oligozoospermia, asthenozoospermia and teratozoospermia) a punctate manner of distribution was seen. (Figure 2A–D).

The capacitated spermatozoa for normospermic men revealed similar pattern of tACE protein expression as ejaculated sperm but with stronger immunofluorescent signals in the neck and mid-piece as well as in post-acrosomal region of the head.

In capacitated sperm from pathological semen samples the reaction area of tACE exhibited punctate pattern of distribution and the intensity of immunofluorescent signals is weaker than the normospermic capaciatetd sperm (Figure 2E–H).

In acrosome reacted sperm of normospermic men tACE immunoreactivity was not visible in acrosomal region and immunofluorescent signals can be seen in equatorial segment and post-acrosomal region of the head. The enzyme is still present in the neck and mid-piece of normospermic and pathological semen samples and the intensity of the fluorescent signals is weaker than capacitated sperm (Figure 2I–L).

**Figure 3:**
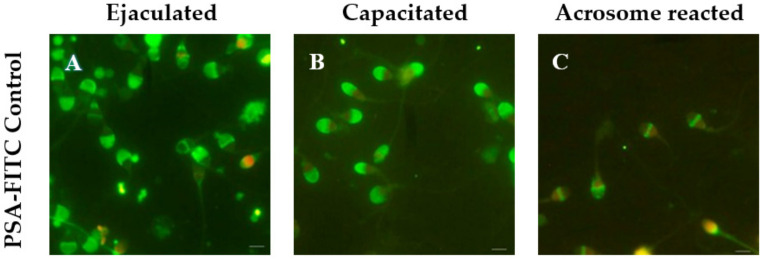
Immunofluorescence reaction for PSA-FITC in ejaculated (**A**), capacitated (**B**) and acrosome-reacted (**C**) spermatozoa. Preservation of the integrity of the acrosome is observed in a large part of the ejaculated and capacitated spermatozoa, while in acrosome-reacted spermatozoa only the equatorial segment is marked. Scale bar = 5 μm.

FITC–PSA staining revealed functional status of sperm during capacitation and acrosome reaction. (Figure 3A–C).

### 3.2. Measurement of Area of tACE Protein Expression in Neck and Mid-Piece Visualized by Immunofluorescence in Different Cases of Male Infertility

**Figure 4:**
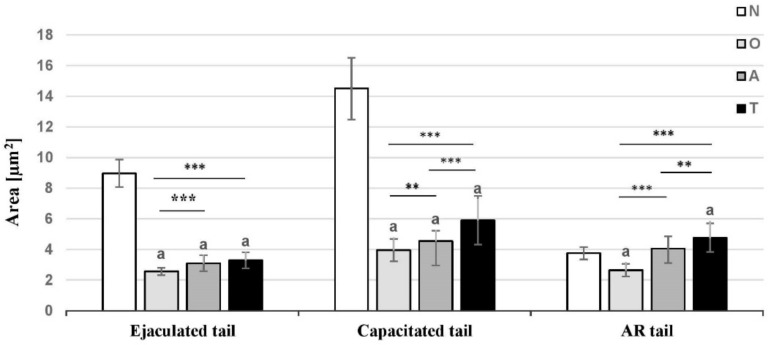
Measurements of area of tACE protein expression in ejaculated, capacitated and acrosome reacted (AR) sperm, visualized by indirect immunofluorescence in different cases of semen pathology N—normospermia, O—oligozoospermia, A—asthenozoospermia, T—teratozoospermia. The data represent mean values ± SD. Asterisk indicates significant differences between groups with semen pathology—** *p* < 0.01; *** *p* < 0.001; a—significance when compared to control value (normospermic men).

Measurements of tACE protein expression area in sperm neck and mid-piece was performed after immunofluorescence done on sperm smears from normospermic and pathological semen samples and the data are presented on Figure 4.

The areas of immunofluorescent signals in tails (neck and mid-piece) of ejaculated and capacitated sperm are significantly reduced in pathological semen samples compared to normospermic ones—by 70% in asthenozoospermia and in oligozoospermia and by 60% in teratozoospermia. The bright area in acrosome reacted tails was significantly lower in oligozoospermic semen samples (by 30%) but not in asthenozoospermic and teratozoospermic samples as compared to normospermic men.

As an attempt to found a trend of changes in tACE area during capacitation and acrosome reaction and the impact of infertile status, we compared the enzyme areas between ejaculated, capacitated and acrosome reacted tails within each group. The values of tACE protein expression area in tails of capacitated sperm were significantly higher than those of ejaculated and acrosome reacted sperm in each group—normospermia, oligozoospermia, asthenozoospermia and teratozoospermia.

However, tACE area of capacitated sperm tails from normospermic men was increased by 62% then that of ejaculated sperm, whereas there was an increase by 47% in astenozoospermic men. The bright area of acrosome reacted tails in normospermia was four times (by 75%) reduced than capacitated ones, whereas in groups with semen pathology there was a decrease of 66% in oligozoospermia, followed by teratozoospermia (by 20%) and asthenozoospermia (by 10%).

In normospermic men tACE protein expression area of acrosome reacted sperm tails was lower by 60% than ejaculated ones. In contrast, the area was higher by 30% and 45% in asthenozoospermic and teratozoospermic men, respectively.

### 3.3. Correlation of tACE Area in Neck and Mid-Piece with Motility and Other Basic Sperm Parameters

**Figure 5:**
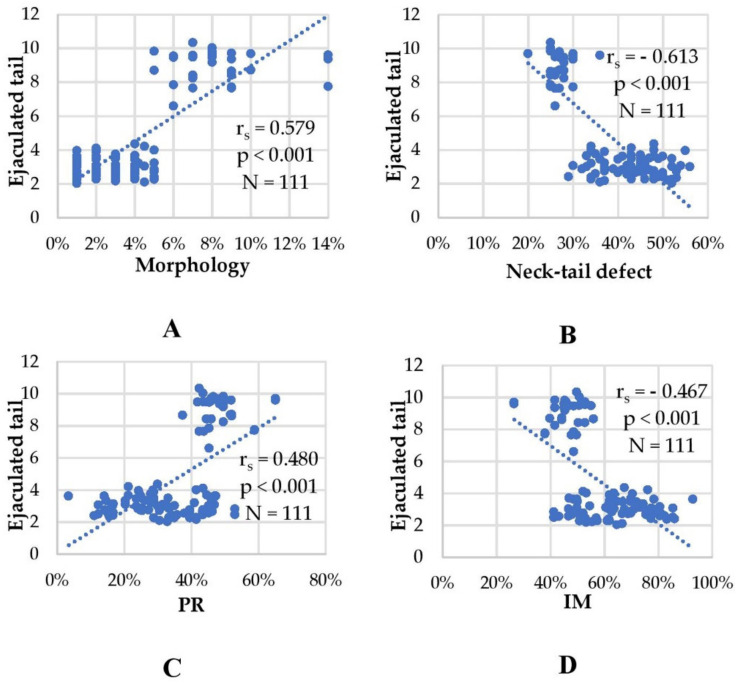
Correlation between the area of expression of tACE protein in the tail (neck and mid-piece) and sperm characteristics of ejaculated spermatozoa. Scatter plots showing the tACE protein expression area in the neck and mid-piece related to percentage of sperm with normal morphology (**A**); percentage of sperm with defect in neck and mid-piece (**B**); percentage of sperm with progressive motility (PR) (**C**); percentage of immotile sperm (**D**). Due to the absence of a normal distribution in most indicators and the heteroscedasticity of the points in the scatter plot, the nonparametric Spearman rank coefficient (r_s_) was used.

To further understand the role of tACE in sperm cells, we analyzed the relationship between basic sperm parameters (concentration, motility, morphology and defects) and the area of tACE protein expression in the tail, we measured on immunofluorescent samples (Figure 5). The tACE bright area in neck and mid-piece was significantly and positively correlated with sperm concentration (r_s_ = 0.509) and level of significance was *p* < 0.01. Significant and positive correlation was also established between tACE positive area and percentage of sperm with normal morphology (r_s_ = 0.579; *p* < 0.01; Figure 5A). Furthermore, we found significant and negative correlation between tACE area and percentage of sperm with defects in neck and mid-piece (r_s_ = −0.613; *p* < 0.01; Figure 5B). Regarding sperm motility, the percentage of progressive spermatozoa was positively correlated with immunofluorescent tACE area (r_s_ = 0.480; *p* < 0.01; Figure 5C), whereas the percentage of immotile sperm showed negative correlation (r_s_ = −0.467; *p* < 0.01; Figure 5D). There was not any significant correlation between the tACE area of protein expression in the tail and the percentage of non-progressive spermatozoa (r_s_ = 0.124; *p* > 0.1).

## 4. Discussion

Molecular studies about sperm physiology could provide new strategies in understanding molecular events leading spermatozoa to achieve fertilization. They could also provide new approaches in the study of factors implicated in male infertility, as routine semen analysis is not able to evaluate fertility potential and hence to predict reproductive outcome [24]. Identification of specific biomarkers is important for precise diagnostic for male infertility and development of adequate therapeutic tools to improve male fertility potential.

Testicular ACE is expressed specifically in developing germ cells and it also present in mature sperm in rodents, domestic animals and humans. In this respect it can be consider as a specific cellular biomarker for fertilization ability of spermatozoa [25]. Our previous studies suggested that tACE can be used as a novel marker for evaluation of germ cell loss and inhibition of spermatogenesis [26]. In human studies, the amount of sperm-bound tACE is more consistent and it appears as a specific marker for different cases of male infertility than amount of sACE in seminal plasma [15]. However, establishment of a possible link between expression/distribution of tACE on sperm surface in different cases of semen pathology and functional characteristics of spermatozoa is the subject of the current research.

The present study provides new data about the localization and distribution of tACE in the tail (neck and mid-piece) in different cases of semen pathology diagnosed according to the WHO criteria (oligozoospermia, asthenozoospermia and teratozoospermia). The tACE protein expression area significantly decreased in sperm form pathological semen samples. Differences in dynamic of the area of tACE protein expression during sperm capacitation and acrosome reaction was found between normal and pathological conditions. Positive correlation was established between tACE area and progressive motility, sperm concentration and normal morphology, whereas the correlation was negative with immobility and defects in sperm neck and mid-piece.

Our data revealed that tACE protein was distributed uniformly, in the neck and mid-piece of ejaculated spermatozoa from normospermic men. Uniform distribution of tACE protein in post-acrosomal region, neck and mid-piece of intact spermatozoa was observed by Nikolaeva et al. (2005 [22] using own generated monoclonal antibodies. Clumpy appearance of the immunofluorescent signals, responsible for punctate manner of distribution in sperm from pathological semen samples was observed for the first time in the current study.

We found that in normospermia the immunoreactivity of tACE protein in the neck and mid-piece of spermatozoa increased during capacitation followed by decrease in acrosome reacted sperm. Post-testicular maturational changes in expression of tACE protein during capacitation were reported by Kohn et al. (1998 [13]) in human normal samples and by Ojaghi et al. (2016 [27]) in bovine sperm, both demonstrated by immunofluorescence. Data about localization and distribution of tACE in capacitated and acrosome reacted sperm in different cases of semen pathology are reported for the first time in the current study.

Our novel quantitative studies involved measurements of the area of tACE protein expression in the neck and mid-piece visualized by immunofluorescence. We found a significant reduction in the bright area in ejaculated and capacitated sperm from pathological semen samples (oligozoospermia, asthenozoospermia and teratozoospermia) compared to normospermia. Absence of tACE expression is responsible for infertility in patients with Total Fertilization Failure and Lower Fertilization Rates [23]. These data suggest importance of tACE for making decision for application of intracytoplasmic sperm injection, not for IVF. The data of the current study could be interpreted in tandem with our previous finding [15] of increased amount of membrane bound tACE (measured by ELISA) in the same pathological conditions. A possible explanation could be found in distributional changes in tACE protein expression in the neck and mid-piece in sperm from pathological semen samples. Normal pattern of uniform/regular distribution of tACE on sperm membrane is necessary for partial release of tACE from sperm membrane, required for fertilizing ability of spermatozoa [22]. The punctate manner of distribution of tACE, found by us, is manifested by decreased area of enzyme expression in semen pathology groups. This finding possibly represents aggregation of tACE complexes that could prevent the normal release of tACE form sperm membrane resulting in increased amount of the membrane bound enzyme, measured by ELISA. According to Kohn et al., 1998; Shibahara et al., 2001 [13,14], the elevation of tACE enzymatic activity is indicative for abnormal retention of the enzyme due to compromised release form sperm membrane during maturation and capacitation.

Testicular ACE was suggested by many authors to play a crucial role in acquisition of fertilizing competence of spermatozoa that involves capacitation/ability for binding to zona pellucida, acrosome reaction and sperm–egg fusion. The enzyme is known to govern both GPI-anchored protein release and lipid raft movement being the main component of sperm surface remodeling machinery [28] that occurred upon capacitation followed by acrosome reaction. Testicular ACE interacts with several essential factor in sperm binding cascade (ADAM 3, Calsperin, Calmegin, Testis Specific Protein Disulphide Isomerase), and their proper localization and distribution on sperm surface is essential for the binding ability of spermatozoa [29,30,31]. A putative target for GPI-ase activity of tACE, necessary for sperm–egg fusion is transmembane protein IZUMO of lipid raft [32]. The knockout models for above mentioned factors exhibited similar phenotype of male infertility like ACE knockout mice [33]. Having in mind complex interaction of tACE with these factors it seems that proper localization and distribution of the enzyme on the sperm membrane could be consider as an essential requirement for successful performance of its main role in fertilization.

For the first time different trends was established in the dynamic of tACE protein expression area in sperm tails (neck and mid-piece) of asthenozoospermic men compared to teratozoospermic and oligozoospermic ones. In particular, tACE area increased by 62% during capacitation in normospermia, whereas in asthenozoospermia and oligozoospermia it was an increase by 47% and 54%, respectively. Considering that sperm cells do not produce proteins de novo, the increase in tACE expression area could be explain by possible uncovering of epitopes recognizable by the antibody due to conformational changes in tACE molecule in the process of remodeling of the sperm membrane during capacitation.

During the acrosome reaction the tACE area decreased to a higher extent in normospermia (by 75%) as compared to groups with semen pathologies (by 10% and 20% in asthenozoospermia and teratozoospermia, respectively). Maturational dynamic in tACE area during capacitation and acrosome reaction was more affected in asthenozoospermia compared to teratozoospermia and oligozoospermia. And the asthenozoospermic sperm has the lowest motility and highest immobility within the infertile group. Asthenozoospermia was one of the leading causes of infertility failure because immotile spermatozoa were unable to reach the oocyte and penetrate normally [34].

In the current study we established positive correlation between tACE protein expression area in the neck and mid-piece with progressive motility of spermatozoa. Negative correlation was established between sperm immobility and defects in neck and mid-piece. Therefore, the tACE expression area in this region probably reflects mobility status of sperm and it could be suggested as novel marker for evaluation of sperm motility. Similar correlations with sperm motility have been observed for levels of Angiotensin II type 2 receptor and they were lower in asthenozoospermic men [34]. Angiotensin II stimulates sperm motility in vitro via Angiotensin II type 1 receptor [35]. A positive relationship was reported between sperm motility and fertilization success [36]. Thus, the application of tACE as a biomarker can be helpful for selection of the most motile spermatozoa for IVF.

There is some discrepancy about correlation of tACE with sperm motility that could be explained by different protocols applied in the assay for measurements of tACE. Briefly, negative correlation was reported between ACE enzyme activity and sperm motility in human [13,14]. The amount of membrane bound tACE, measured by ELISA also correlate negatively with sperm motility [15]. The levels of tACE measured by flow cytometry correlate in opposite manners with sperm motility depending on the sperm samples used [16,37].

## 5. Conclusions

Nevertheless, data clearly indicated clinical significance of evaluation tACE expression on human spermatozoa for diagnostics of male infertility. The development of adequate assays of tACE expression on the surface of spermatozoa is important for future research on the role of tACE in male fertility.

## Figures and Tables

**Table 1:** Semen analysis and the main sperm characteristics in normosperminc (N) and infertile men (A—asthenozoospermia; O—oligozoospermia and T—teratozoospermia).

	N	A	O	T
	*n* = 31	*n* = 31	*n* = 25	*n* = 24
Sperm concentration 10^6^/mL	49.37 ± 31.44	32.73 ± 14.60 * p = 0.011	11.58 ± 2.42 *** p = 0.000	37.32 ± 17.99 ns *p* = 0.080
Count, × 10^6^	88.33 ± 54.01	82.37 ± 72.41 ns *p* = 0.715	20.81 ± 12.30 *** p = 0.000	60.83 ± 53.81 ns *p* = 0.066
Progressive sperm motility %	48.46 ± 6.36	25.35 ± 5.88 *** p = 0.000	34.95 ± 12.01 *** p = 0.000	30.48 ± 13.54 *** p = 0.000
Non progressive sperm motility %	5.86 ± 2.45	5.69 ± 2.78 ns *p* = 0.791	5.99 ± 2.46 ns *p* = 0.848	4.50 ± 1.90 * p = 0.028
Immotile sperm %	45.68 ± 6.88	68.64 ± 6.44 *** p = 0.000	58.46 ± 13.15 *** p = 0.000	64.61 ± 13.57 *** p = 0.000
Morphology %	8.26 ± 2.32	3.41 ± 1.46 *** p = 0.000	2.52 ± 0.96 *** p = 0.000	1.75 ± 0.79 *** p = 0.000
Head defect %	83.13 ± 1.69	93.35 ± 1.80 *** p = 0.000	95.96 ± 1.86 *** p = 0.000	97.25 ± 1.57 *** p = 0.000
Tail (neck and mid-piece) defect %	27.16 ± 2.72	39.06 ± 5.29 *** p = 0.000	45.36 ± 4.83 *** p = 0.000	47.17 ± 5.44 *** p = 0.000

* *p* < 0.05; *** *p* < 0.001; ns—not significant. Note: *p* = Sig. (2-tailed) from the test of hypothesis: H_0_: $\bar{X}$_N_ = $\bar{X}$_A_, respectively H_0_: $\bar{X}$_N_ = $\bar{X}$_O_ and H_0_: $\bar{X}$_N_ = $\bar{X}$_T_.

## Data Availability

The data presented in this study are available on request from the corresponding author. The data are not publicly available due to privacy and ethical restrictions related to the patient informed consent.
